# Editorial: Apicomplexa Epidemiology, Control, Vaccines and Their Role in Host-Pathogen Interaction

**DOI:** 10.3389/fvets.2022.885181

**Published:** 2022-03-29

**Authors:** Hany M. Ibrahim, Valeria A. Sander

**Affiliations:** ^1^Immunology and Physiology Unit, Zoology Department, Faculty of Science, Menoufia University, Shebin El-Kom, Egypt; ^2^Laboratorio de Molecular Farming y Vacunas, INTECH, CONICET-UNSAM, Chascomús, Argentina

**Keywords:** apicomplexa, epidemiology, control, vaccines, interactions

Apicomplexan parasites are protozoa recognized as the causative agents of threatening diseases both of human and veterinary relevance, including several important species from the genera *Toxoplasma, Sarcocystis, Plasmodium, Neospora, Eimeria, Cryptosporidium, Theileria*, and *Babesia* (1, 2). They have complicated life cycles which are characterized by three distinguished processes: sporogony, merogony, and gametogony (1). These parasites possess an apical complex that participates in the invasion of the host cell and contains rhoptries, micronemes, polar rings, and conoid (1, 2). Annotation and analysis of the genomes from different species within the group have demonstrated surprising variations in the biology of Apicomplexa, in particular in some key metabolic pathways (1, 2).

The high incidence of infection by these parasites is associated with a high burden of disease and causes also important economic losses. Despite their relevance, few vaccines and chemotherapeutic drugs are available against Apicomplexan parasites; a state of affairs this is possible to grow to be worse with the emergence of recent resistant strains of parasites. Therefore, to set up the foundation for control programs, many trials related to host-parasite interaction, vaccine development, the discovery of drug targets, genome sequences, genes expression, characterization, and proteomic analysis have emerged. The major challenge is how to take advantage of that information to derive insights into parasite virulence, its capacity to direct the host response and become aware of unique genes, proteins, and molecules that constitute the best amenable targets for vaccine development, and the discovery of drug targets.

The aim of this Research Topic was to bring together recent epidemiological data about apicomplexan infections and novel approaches to identify new molecular targets to discuss biological insights that have emerged as a consequence of their analysis. Of particular interest are potential anti-parasitic vaccines and drug targets, as well as studies on host-parasite interaction that potentially would open up new opportunities to discover novel therapeutic approaches. In this special e-collection there are 8 papers covering the above mentioned aspects.

Identification and discovery of new genes from these parasites, their impact on host-parasite interaction and their potential as vaccine candidates has been the most commonly evaluated aspect. Four papers out of 8 (50%) were concerned with this topic, which represents one of the most important steps in discovering novel therapeutic approaches. Data from Gao et al. showed that one of the three *Cryptosporidium parvum* rhomboid peptidases, *C. parvum* rhomboid membrane protein 1 (CpROM1), an intra-membrane peptidase with membrane proteolytic activity, is involved in host–parasite interactions, including invasion and proteostasis of parasitophorous vacuole membranes, and feeder organelles. Also identification and characterization of recombinant *Babesia divergens* P0 protein (rBdP0) indicates the possible diagnostic use of rBdP0 and recommends this antigen to serve as a vaccine candidate against babesiosis in animals or humans (El-Sayed et al.). Tian et al. demonstrated that *Toxoplasma gondii* perforin-like protein 2 (TgPLP2) that contains a membrane attack complex/perforin domain can enhance both humoral and cellular immune responses to protect host against toxoplasmosis and thus, it represents a potential candidate for *T. gondii* vaccines. The group of Ozubek identified and characterized two novel non-canonical members of the CCp gene family in *Babesia bovis*, named CCp5 and FNPA and their data suggest that FNPA can be used as a possible target of a transmission-blocking vaccine against *B. bovis*.

Moreover, related to the previously mentioned topic, Tomazic et al. displayed in their review a comprehensive summary of advances in “omics,” CRISPR/Cas9 technologies and systems biology approaches applied to apicomplexan parasites of economic and zoonotic importance, highlighting their potential in future vaccine development.

The detection, occurrence, and molecular identification of these parasites constitute another important issue in this topic. Two papers out of 8 (25%) were related to this aspect. The study of Jiang et al. demonstrated the first detection of *Sarcocystis* spp. infection in a captive alpaca (*Vicugna Pacos*) from China which showed a high similarity to *Sarcocystis masoni* by using sequence analysis. The work of Máca et al. provided the first record on *Sarcocystis* from owls obtained in the Kauhava region of west-central Finland.

Finally, Mesa-Pineda et al. reviewed the basic concepts of coccidiosis, the various *Eimeria* species which infect chickens, their life cycle, and the most sustainable and holistic methods available to control this disease.

In summary, the data of the above mentioned studies and reviews represent an enormous amount of new relevant data on the Apicomplexa occurrence and control. Finally, this editorial article that summarizes what has been mentioned in this special issue considers a step in the way to consolidate the knowledge of this group of parasites in order to reach effective ways to control it.

## Author Contributions

HI wrote the first version of the manuscript. VS revised the manuscript. All authors contributed to the article and approved the submitted version.

## Conflict of Interest

The authors declare that the research was conducted in the absence of any commercial or financial relationships that could be construed as a potential conflict of interest.

## Publisher's Note

All claims expressed in this article are solely those of the authors and do not necessarily represent those of their affiliated organizations, or those of the publisher, the editors and the reviewers. Any product that may be evaluated in this article, or claim that may be made by its manufacturer, is not guaranteed or endorsed by the publisher.
